# Epicatechin Gallate Blocks GC/GR Signaling to Suppress Stress-Induced Myeloid Differentiation of HSPCs and Subsequent TNBC Metastasis

**DOI:** 10.3390/ph19081211

**Published:** 2026-08-01

**Authors:** Meiling Ma, Guanzhi Li, Qin Xu, Guangxian Zhang, Chuanjun Shen, Zhitao Guo, Xuezhen Li, Yifeng Zheng, Shengqi Wang, Bo Pan, Juping Zhang, Yaxiao Liu, Jianping Chen, Zhiyu Wang, Cheng Peng, Neng Wang

**Affiliations:** 1 State Key Laboratory of Traditional Chinese Medicine Syndrome, Research Centre of Basic Integrative Medicine, School of Basic Medical Sciences, Guangzhou University of Chinese Medicine, Guangzhou 510006, China; 2 State Key Laboratory of Southwestern Chinese Medicine Resources, Chengdu University of Traditional Chinese Medicine, Chengdu 611137, China; 3 Guangdong Provincial Key Laboratory of Clinical Research on Traditional Chinese Medicine Syndrome, Guangdong Provincial Academy of Chinese Medical Sciences, The Second Affiliated Hospital of Guangzhou University of Chinese Medicine, Guangzhou 510006, China; 4 Department of Physiotherapy, School of Acupuncture and Rehabilitation Medicine, Guangzhou University of Chinese Medicine, Guangzhou 510006, China; 5 Guangdong Provincial Key Laboratory of Research and Development in Traditional Chinese Medicine, The Fifth Clinical College, Guangzhou University of Chinese Medicine, Guangzhou 510006, China; 6 School of Chinese Medicine, LKS Faculty of Medicine, The University of Hong Kong, Hong Kong, China

**Keywords:** TNBC, chronic psychological stress, ECG, GC/GR axis, MDSCs, HSPCs

## Abstract

**Background:** Chronic psychological stress drives metastasis in triple-negative breast cancer (TNBC), yet the underlying mechanisms remain poorly understood and effective interventions are lacking. Stress-induced expansion of myeloid-derived suppressor cells (MDSCs) and subsequent immune remodeling play critical roles, with aberrant myeloid differentiation of hematopoietic stem and progenitor cells (HSPCs) serving as a major source of MDSCs. This study investigates whether epicatechin gallate (ECG) suppresses stress-driven TNBC growth and lung metastasis by regulating HSPC myeloid differentiation. **Methods:** A mouse model of chronic unpredictable mild stress (CUMS) followed by 4T1 tumor implantation was used to evaluate the anti-tumor effects of ECG. CETSA-WB, molecular docking, HSPC differentiation assays, and MDSC functional validation assays, along with immunohistochemistry, immunofluorescence, and flow cytometry, were performed to elucidate how ECG modulates glucocorticoid (GC)/glucocorticoid receptor (GR) signaling and HSPC differentiation. **Results:** ECG dose-dependently alleviated depressive-like behaviors, reduced serum corticosterone (Cort), and inhibited tumor growth and lung metastasis. Notably, ECG decreased lung metastatic foci by 76.9% relative to the CUMS group. Mechanistically, chronic stress activated GR and induced its nuclear translocation in HSPCs, promoting aberrant HSPC-to-MDSC differentiation. ECG directly bound GR, blocked its nuclear translocation, and suppressed the myeloid differentiation of HSPCs into MDSCs, which was accompanied by downregulation of S100A8/A9, fibronectin, and MMP-2, as well as increased CD8^+^ T cell infiltration. Supernatants from ECG-pretreated and differentiated HSPCs reversed Cort-induced epithelial–mesenchymal transition (EMT) in 4T1 cells. **Conclusions:** ECG acts as a natural GR signaling blocker that directly targets GR to block chronic stress-driven abnormal myeloid differentiation of HSPCs, thereby remodeling the pulmonary immune microenvironment, suppressing EMT, and reducing breast cancer lung metastasis. These findings identify ECG as a promising GR signaling blocker and a potential adjuvant therapy for cancer patients under high-stress conditions.

## 1. Introduction

Triple-negative breast cancer (TNBC) accounts for 15–20% of all breast cancer cases and lacks expression of estrogen receptor (ER), progesterone receptor (PR), and human epidermal growth factor receptor 2 (HER2) [1]. This tumor type is highly aggressive and prone to early metastasis. Recurrence and distant metastasis represent persistent clinical hurdles; indeed, 30–50% of high-risk patients develop relapse or visceral metastasis after standard treatment [2], and once relapsed, median overall survival (mOS) is less than one year, reflecting an exceptionally poor prognosis in breast cancer [3]. Studies have confirmed that chronic psychological stress is an important exogenous risk factor that promotes the initiation, progression, and distant metastasis of breast cancer. Clinical data indicate that depression is associated with a 24% higher risk of breast cancer recurrence, a 29% higher risk of breast cancer-specific mortality, and comorbid depression and anxiety are associated with a 45% higher risk of breast cancer-specific mortality [4]. Mechanistically, chronic psychological stress persistently activates the hypothalamic–pituitary–adrenal (HPA) axis and the sympathetic nervous system (SNS), leading to substantial release of stress hormones and neurotransmitters, including glucocorticoids (GCs) and catecholamines [5]. Among these, GCs serve as core mediators of the stress response. They not only act directly on tumor cells to activate downstream pro-tumorigenic signaling but also increase tissue sensitivity to catecholamines through their classical permissive effect, synergistically amplifying the pro-cancer effects of stress signals [6,7,8]. The chronic unpredictable mild stress (CUMS) paradigm is a well-established rodent model for inducing HPA axis activation and sustained GC elevation, and has been extensively used to investigate the regulatory role of stress in disease progression. Nevertheless, research focusing on how chronic stress remodels the tumor immunosuppressive microenvironment to indirectly promote tumor metastasis remains limited, highlighting a critical area for in-depth investigation in stress-related oncology.

Although TNBC is more responsive to immunotherapies owing to its immunogenicity, this therapeutic vulnerability can be undermined by chronic psychological stress, which promotes an immunosuppressive tumor microenvironment (TME)—a key driver of immune evasion and distant metastasis. This TME is often characterized by aberrant expression of key molecules such as S100 calcium-binding protein A8/A9 (S100A8/A9), fibronectin, and matrix metalloproteinase-2 (MMP-2), as well as alterations in CD8^+^ T cells-all of which are closely associated with the function of myeloid-derived suppressor cells (MDSCs). Among these, S100A8/A9 directly drives the differentiation and accumulation of MDSCs by binding to the receptor for advanced glycation end-products/toll-like receptor 4 (RAGE/TLR4) receptors and activating downstream signaling pathways to promote their expansion [9]. Fibronectin regulates the differentiation and immunosuppressive function of MDSCs, and its deficiency significantly reduces MDSC accumulation [10]. MMP-2 is involved in regulating the ability of MDSCs to migrate to peripheral sites, thereby enhancing their recruitment into the tumor microenvironment [11]. MDSCs and CD8^+^ T cells interact in complex ways, with MDSCs suppressing antitumor CD8^+^ T cell function through multiple mechanisms. For instance, activated MDSCs induce metabolic dysfunction, proliferative arrest, and exhaustion in CD8^+^ T cells via L-arginine depletion, production of reactive oxygen and nitrogen species, and Programmed death-ligand 1 (PD-L1) upregulation [12], thereby creating an immunosuppressive microenvironment that promotes tumor growth and metastasis. The aforementioned molecules collectively constitute a critical network that regulates the fate of MDSCs. Studies have shown that MDSCs in tumor-bearing mice were cleared significantly faster than normal myeloid cells and exhibited very poor survival after overnight culture in vitro, indicating that MDSCs are inherently short-lived. As such, maintaining peripheral MDSC numbers depends on continuous replenishment from bone marrow-derived hematopoietic stem and progenitor cells (HSPCs), which undergo sustained proliferation and differentiation to compensate for rapid MDSC apoptosis in peripheral tissues [13]. Research indicates that chronic psychological stress induces GC/glucocorticoid receptor (GR) axis activation, positively regulating HSPCs, markedly elevating their numbers, whereas GR deficiency attenuates HSPC counts [14]. Nevertheless, how GR-mediated signaling regulates myeloid differentiation of HSPCs in tumors remains poorly understood, and its connection to stress-induced immunosuppression in the tumor microenvironment and subsequent metastasis is still unclear.

Natural small molecules demonstrate substantial antitumor biological activities, and their antitumor effects are characterized by multi-target and multi-stage properties, rendering them a research focus in the domain of natural products for targeted intervention in the progression of malignant tumors. Epicatechin gallate (ECG) is an important naturally occurring polyphenolic bioactive compound widely found in green tea, white tea, oolong tea, and other tea varieties [15]. It is also present in Traditional Chinese Medicine (TCM) with antitumor and immunomodulatory properties, such as *Sanguisorba officinalis* and *Spatholobus suberectus*. The direct anti-tumor effects of ECG against melanoma, prostate cancer, pancreatic cancer, and other tumors have been extensively reported [16,17,18]. ECG exhibits diverse anticancer mechanisms, including downregulation of key lipid metabolism enzymes (acetyl-CoA carboxylase (ACC), ATP-citrate lyase (ACLY), fatty acid synthase (FASN)) and the phosphatidylinositol 3-kinase/protein kinase B/mammalian target of rapamycin (PI3K/AKT/mTOR) pathway to impair lipid synthesis and cell migration [17], as well as reversal of transforming growth factor-β1 (TGF-β1)-induced EMT, inhibition of MMP-2/urokinase plasminogen activator (uPA) and invasion, and anti-metastatic effects through stromal remodeling and matrix degradation [19].

Despite these advances, a critical knowledge gap remains: can ECG directly target the GR signaling pathway to block chronic stress-driven abnormal myeloid differentiation of HSPCs and thereby inhibit TNBC metastasis? To address this question, the present study was designed to explore the intervention effect of ECG on TNBC proliferation and metastasis promoted by the chronic stress-mediated immunosuppressive microenvironment, to clarify the mechanism by which ECG directly targets GR and inhibits its nuclear translocation to reverse the abnormal myeloid differentiation bias of HSPCs, and to reveal the key role of ECG in regulating EMT through the GR-HSPC axis. By integrating chronic unpredictable mild stress (CUMS) tumor-bearing models, cellular thermal shift assay coupled with Western blot (CETSA-WB) assay, molecular docking and HSPC differentiation assay, as well as MDSC functional validation assay, we uncovered a novel function of ECG in inhibiting stress-induced myeloid differentiation and TNBC metastasis, thereby identifying ECG as a promising GR signaling blocker for cancer patients under high-stress conditions.

## 2. Results

### 2.1. ECG Inhibits Chronic Stress-Induced Breast Cancer Growth

**Figure 1:**
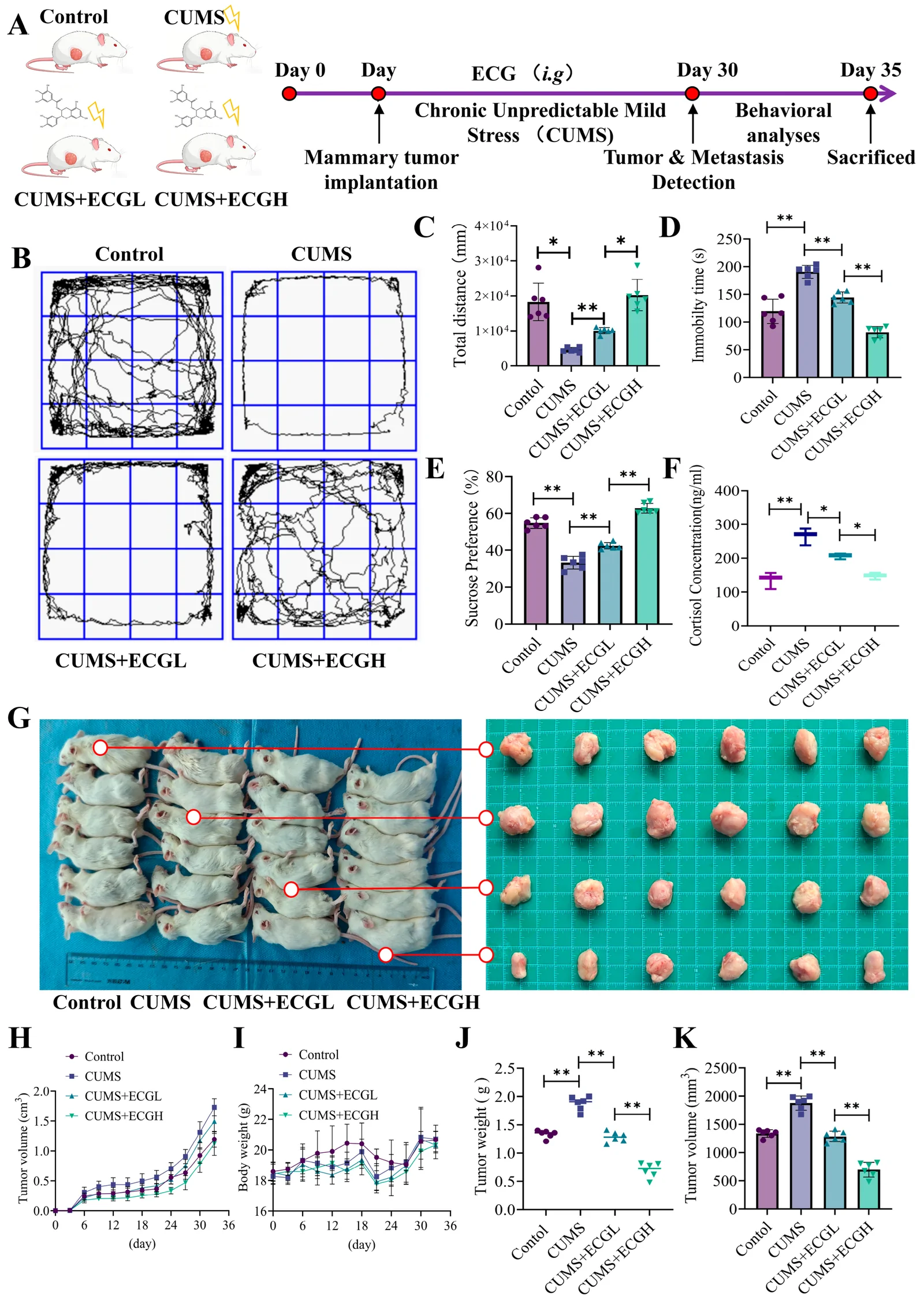
Epicatechin gallate (ECG) inhibits chronic stress-induced primary breast cancer growth. Mice were subjected to chronic unpredictable mild stress (CUMS) and treated with vehicle, low-dose ECG (ECGL, 25 mg/kg), or high-dose ECG (ECGH, 50 mg/kg). (**A**) Schematic diagram of the animal experimental timeline. (**B**–**E**) Behavioral assessments (*n* = 6): (**B**) representative traces of the open field test; (**C**) total distance traveled in the open field test; (**D**) immobility time in the forced swim test; (**E**) sucrose preference in the sucrose preference test. (**F**) Serum corticosterone levels (*n* = 3). (**G**–**K**) Tumor growth (*n* = 6): (**G**) representative images of mice (*left*) and excised tumors (*right*); (**H**) Statistical analysis of primary tumor growth curves, (**I**) body weight curves, (**J**) tumor weight and (**K**) tumor volume. Data are presented as the mean ± standard deviation (SD). * *p* < 0.05, ** *p* < 0.01.

This study aimed to determine the in vivo effects of ECG on chronic stress-promoted breast cancer progression. A CUMS model was established in BALB/c mice to systematically evaluate the regulatory effects of ECG on tumor growth and metastasis. Four groups were included: Control group, CUMS group, CUMS + low-dose ECG (ECGL) group (25 mg/kg), and CUMS + high-dose ECG (ECGH) group (50 mg/kg). BALB/c mice were inoculated with 4T1 cells on day 7 of model establishment. They then received daily intragastric administration and CUMS treatment. This continued until the endpoint. At the endpoint, behavioral assessments were performed and tumor growth was evaluated (Figure 1A).

The behavioral assessments revealed that CUMS mice, compared to controls, had a shorter central area residence time (*p* < 0.05; Figure 1B,C). Their immobility time was longer (*p* < 0.01; Figure 1D). Their sucrose preference was lower (*p* < 0.01; Figure 1E). These findings confirmed successful CUMS modeling. ECG administration reversed these depressive-like behaviors, with greater efficacy at the high dose. Serum cortisol Enzyme-Linked Immunosorbent Assay (ELISA) detection further revealed that chronic stress elevated cortisol levels (*p* < 0.01), which were dose-dependently reduced by ECG (*p* < 0.05, Figure 1F), suggesting that ECG’s behavioral benefits may involve cortisol regulation.

Tumor growth analysis demonstrated that chronic stress significantly promoted orthotopic tumor growth compared with the Control group. Representative images of mice and their dissected tumors at the endpoint revealed intergroup differences in tumor size (Figure 1G). Dynamic monitoring showed that tumor volumes in the CUMS group began to increase markedly from day 18 post-inoculation, and this difference persisted until the endpoint. ECG intervention dose-dependently suppressed stress-induced tumor growth (Figure 1H). No significant differences in body weight were observed among the groups, suggesting that ECG has favorable biosafety (Figure 1I). At the experimental endpoint, both tumor weight and volume were significantly higher in the CUMS group than in the Control group (Figure 1J,K, *p* < 0.01).

### 2.2. ECG Inhibits Chronic Stress-Induced Breast Cancer Lung Metastasis

**Figure 2:**
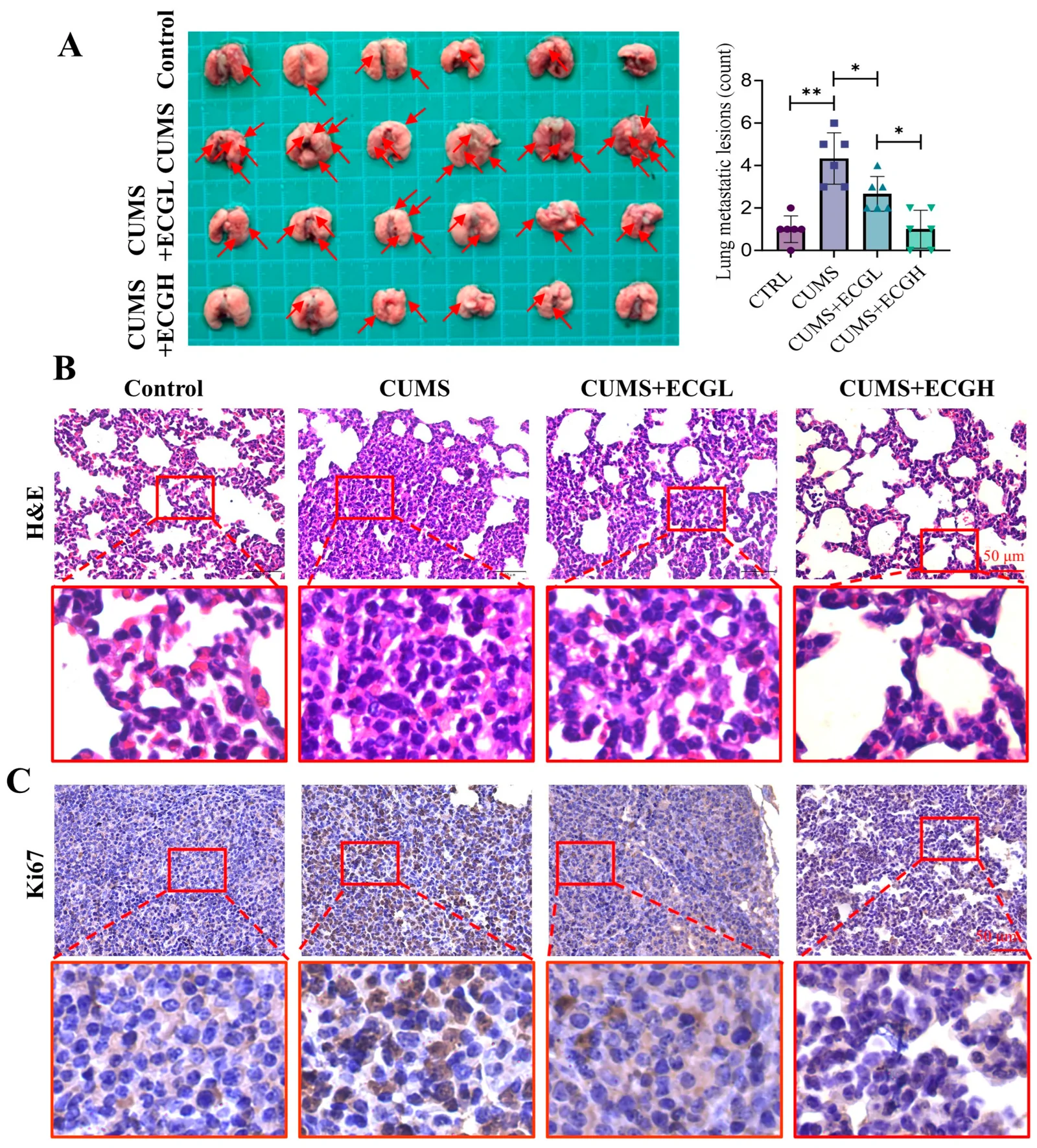
Epicatechin gallate (ECG) inhibits chronic stress-induced breast cancer lung metastasis. Mice were subjected to chronic unpredictable mild stress (CUMS) and treated with vehicle, low-dose ECG (ECGL, 25 mg/kg), or high-dose ECG (ECGH, 50 mg/kg). (**A**) Quantification of metastatic nodules in the lungs (*n* = 6). Arrows indicate lung metastatic foci. Data are presented as mean ± standard deviation (SD). * *p* < 0.05, ** *p* < 0.01. (**B**) Representative hematoxylin and eosin (H&E) staining images of lung metastatic tissues. (**C**) Representative images of Ki67 immunohistochemistry in lung metastatic tissues. Scale bar: 50 μm.

Compared with the Control group, the CUMS group exhibited a significantly increased number of lung metastatic foci (*p* < 0.01), which was markedly reduced by high-dose ECG intervention (*p* < 0.05; Figure 2A). Hematoxylin and eosin (H&E) staining was performed. The results showed that CUMS induced obvious lung pathological damage. The damage included alveolar disruption, inflammatory infiltration, and metastatic foci formation. ECG treatment ameliorated these changes. The tissue structure was restored to near-normal (Figure 2B). Immunohistochemical analysis further showed that CUMS increased the proportion of Ki67-positive cells in lung metastases, an effect reversed by ECG (Figure 2C). Collectively, these findings demonstrate that ECG effectively inhibits chronic psychological stress-induced lung metastasis of breast cancer.

### 2.3. ECG Reverses Stress-Induced Formation of the Pulmonary Immunosuppressive Microenvironment and Expansion of MDSCs to Inhibit Tumor Metastasis

**Figure 3:**
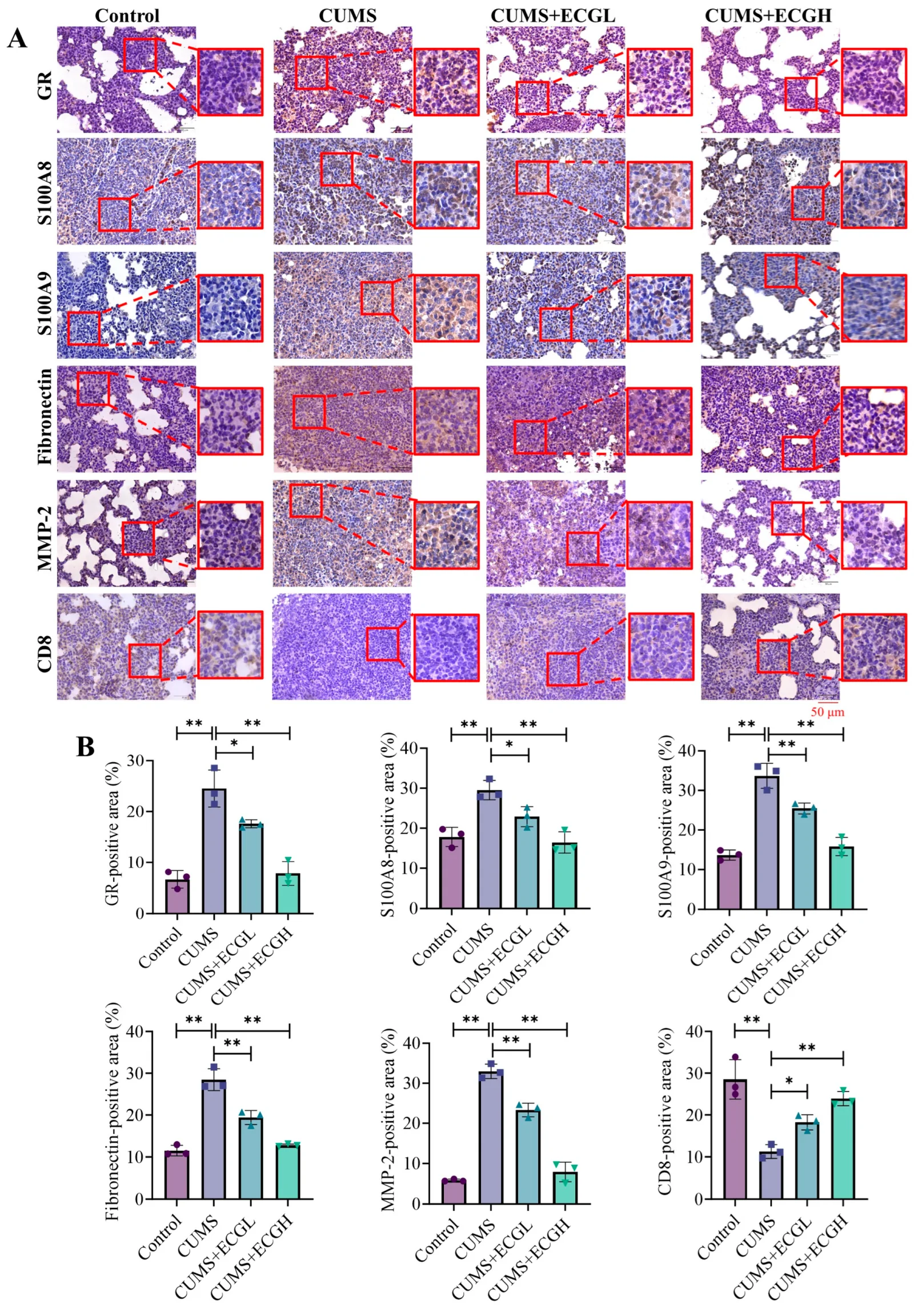
Epicatechin gallate (ECG) reverses stress-induced formation of the pulmonary immunosuppressive microenvironment and expansion of myeloid-derived suppressor cells (MDSCs) to inhibit tumor metastasis. Mice were subjected to chronic unpredictable mild stress (CUMS) and treated with vehicle, low-dose ECG (ECGL, 25 mg/kg), or high-dose ECG (ECGH, 50 mg/kg). (**A**) Representative immunohistochemistry images of glucocorticoid receptor (GR), S100A8, S100A9, fibronectin, matrix metalloproteinase-2 (MMP-2), and CD8 in lung metastatic foci. Scale bar: 50 μm. (**B**) Quantitative analysis of immunohistochemical staining (*n* = 3). Data are presented as the mean ± standard deviation (SD). * *p* < 0.05, ** *p* < 0.01.

**Figure 4:**
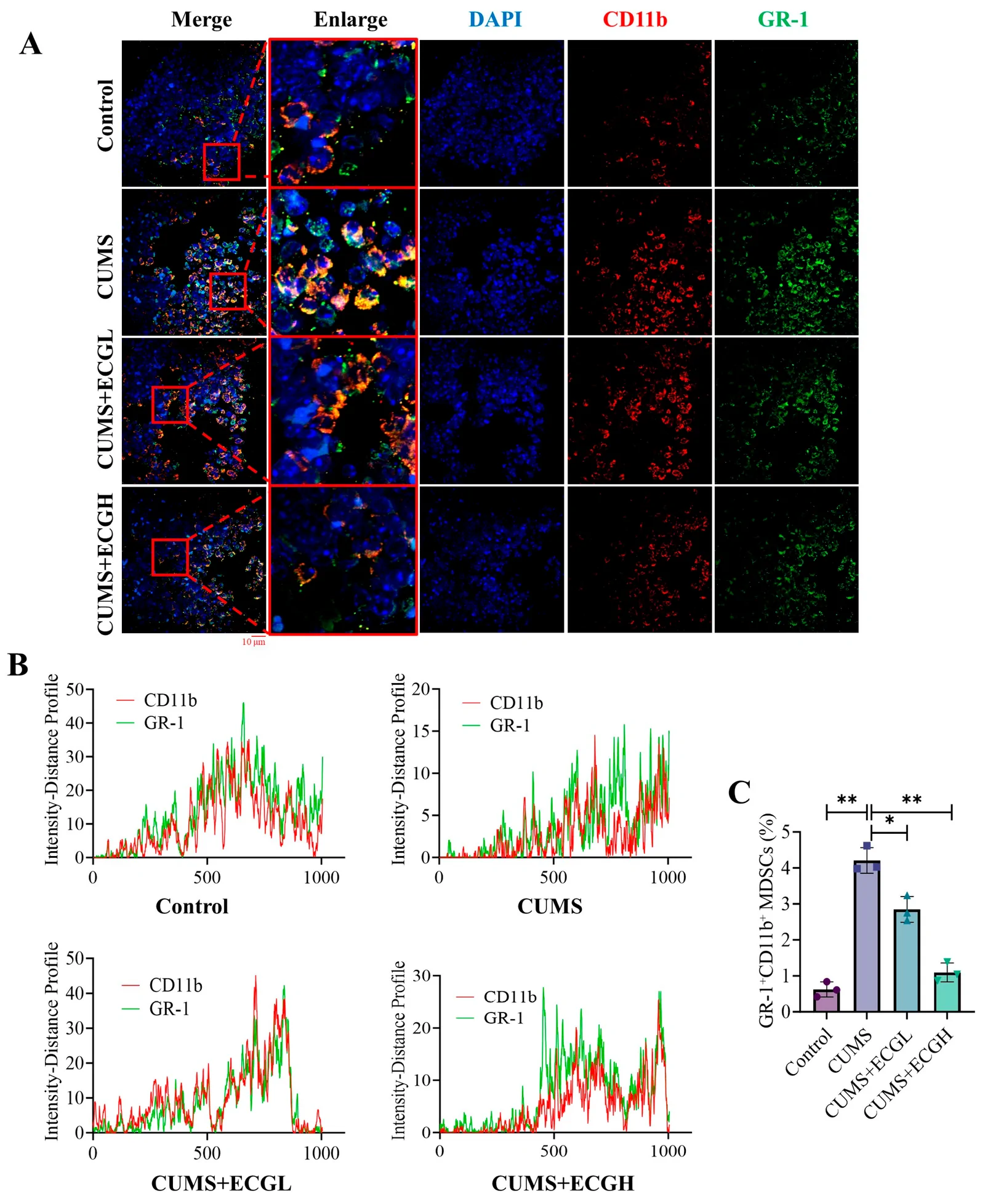
Epicatechin gallate (ECG) reverses stress-induced formation of the pulmonary immunosuppressive microenvironment and myeloid-derived suppressor cells (MDSCs) expansion to inhibit tumor metastasis. Mice were subjected to chronic unpredictable mild stress (CUMS) and treated with vehicle, low-dose ECG (ECGL, 25 mg/kg), or high-dose ECG (ECGH, 50 mg/kg). (**A**) Representative immunofluorescence images of CD11b^+^Gr-1^+^ MDSCs in lung metastatic tissues. Blue: DAPI; Red: CD11b; Green: Gr-1. scale bar: 10 μm. (**B**) Fluorescence colocalization curves. (**C**) Quantitative analysis of CD11b^+^Gr-1^+^ images (*n* = 3). Data are presented as the mean ± standard deviation (SD). * *p* < 0.05, ** *p* < 0.01.

IHC and immunofluorescence analyses were performed on lung tissue sections. Relative to the Control group, the CUMS group showed significantly upregulated GR expression. This occurred in response to GC stimulation. The levels of MDSC-associated markers S100A8 and S100A9 were also elevated. The expression of fibronectin and MMP-2 was increased. These are key mediators of matrix remodeling and tumor invasion. Moreover, CD8 expression was significantly reduced in the CUMS group. These coordinated changes in the expression of the aforementioned molecules collectively established a pulmonary immunosuppressive microenvironment that facilitated tumor metastasis. After ECG intervention, these indicators showed dose-dependent improvements: the expression levels of GR, S100A8, S100A9, fibronectin, and MMP-2 were significantly downregulated, while CD8 expression was partially restored. Such findings imply that ECG effectively reversed the CUMS-induced formation of the pulmonary immunosuppressive microenvironment (Figure 3A,B). To further validate these findings, immunofluorescence staining was performed to detect MDSC infiltration in lung tissues. The results showed that compared with the Control group, the CUMS group exhibited significantly enhanced fluorescence intensity of CD11b^+^Gr-1^+^ MDSCs in lung tissues, with increased overlap of positive signal areas, indicating substantial recruitment and accumulation of MDSCs in the lungs. Compared with the CUMS group, the ECGL group showed reduced fluorescence intensity of MDSCs in lung tissues. In addition, the ECGH group exhibited markedly diminished fluorescence intensity, along with a reduced overlap of fluorescent signal areas. These findings suggest that ECG intervention significantly reduces MDSC infiltration and accumulation in the lungs, restores immune surveillance capacity, and thereby inhibits tumor lung metastasis (Figure 4A–C).

### 2.4. ECG Directly Binds to GR, Inhibits Its Nuclear Translocation, and Blocks the Differentiation of HSPCs into MDSCs

The above-mentioned results verified that breast cancer lung metastasis induced by chronic stress is related to a significant enrichment of MDSCs in the pulmonary metastasis microenvironment. Given the short half-life of MDSCs in vivo, their sustained expansion inevitably depends on the mobilization and differentiation of upstream HSPCs [20]. Therefore, the present study further investigated the central role of HSPCs in chronic stress-promoted MDSC generation and elucidated the regulatory mechanism of ECG in this process.

**Figure 5:**
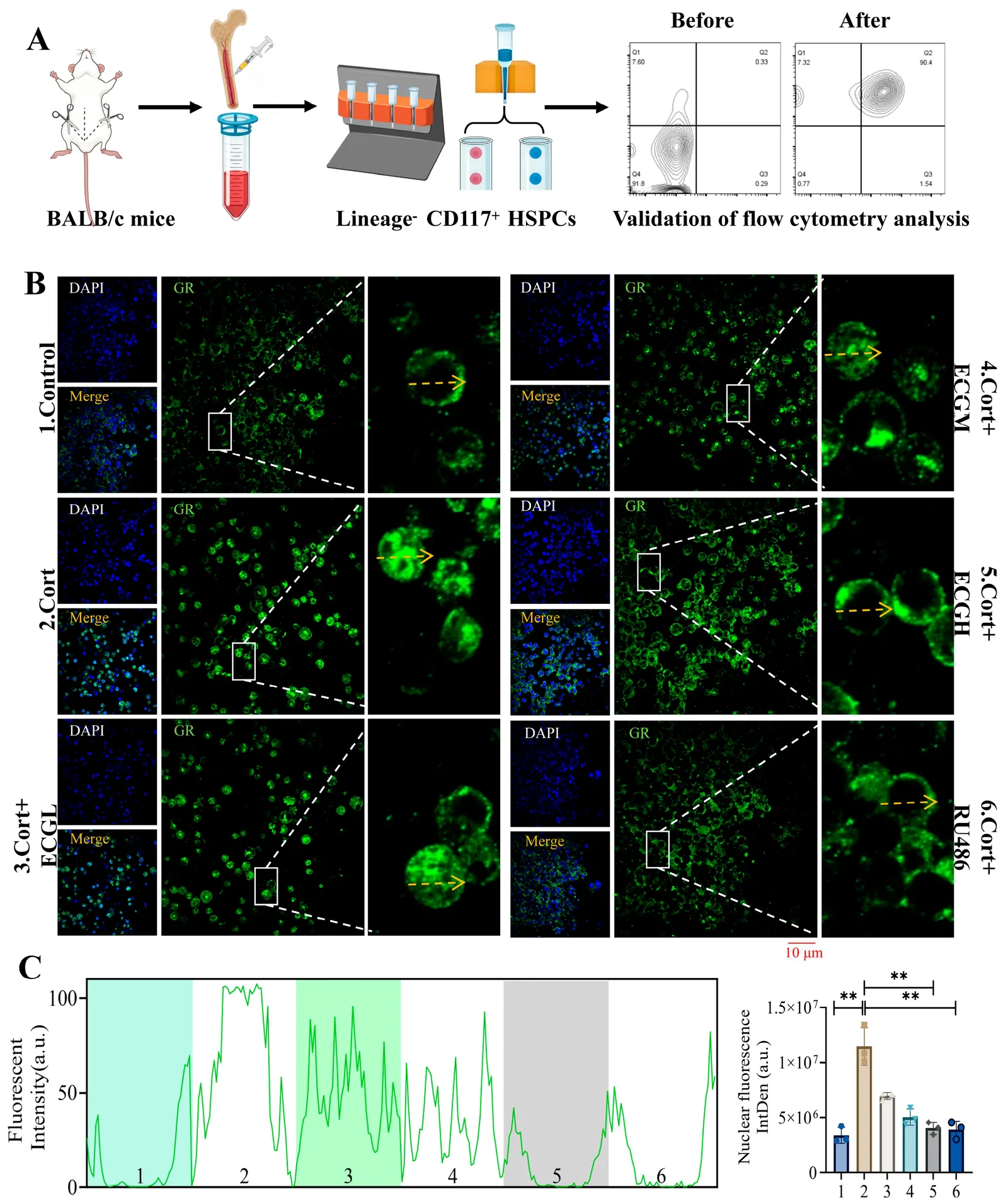
Epicatechin gallate (ECG) directly binds to glucocorticoid receptor (GR), inhibits its nuclear translocation, and blocks the differentiation of hematopoietic stem and progenitor cells (HSPCs) into myeloid-derived suppressor cells (MDSCs) in vitro. HSPCs were treated with 200 nM corticosterone (Cort) in the presence or absence of low-dose ECG (ECGL, 0.1 μM), medium-dose ECG (ECGM, 1 μM), high-dose ECG (ECGH, 10 μM), or RU486 (200 nM) for 24 h. (**A**) Schematic diagram of magnetic-activated cell sorting (MACS) for isolation and analysis of HSPCs. (**B**) Immunofluorescence analysis of GR expression in HSPCs: representative immunofluorescence images of cells treated with 200 nM Cort, different doses of ECG, and RU486. Blue: DAPI; Green: GR. scale bar: 10 μm; (**C**) Left: qualitative fluorescence intensity line-scan profiles along the yellow dashed line and fluorescence intensity quantification. Right: Quantitative analysis of immunofluorescence for each group. Data are presented as the mean ± standard deviation (SD). ** *p* < 0.01.

HSPCs with a lineage-negative, CD117-positive (Lin^−^/c-Kit^+^/Sca-1^+^) phenotype were isolated from mouse bone marrow using magnetic-activated cell sorting (MACS) combined with flow cytometry analysis for subsequent experiments (Figure 5A). Cort exerts its biological effects primarily through binding to GR. Ligand binding triggers a conformational change in GR, leading to its nuclear translocation, where it regulates transcription of downstream target genes [21,22]. Under basal conditions without Cort stimulation, GR nuclear translocation showed no obvious changes across groups, indicating ECG does not obviously affect basal GR localization (Supplementary Figure S1A). Upon Cort treatment, GR fluorescence intensity and nuclear accumulation increased, indicating GR upregulation and nuclear translocation. However, ECG intervention reversed these effects in a dose-dependent manner. In the Cort + ECGL group, overall GR fluorescence intensity decreased versus the Cort group, with lessened nuclear translocation and partial cytoplasmic retention. In the Cort + medium-dose ECG (EGCM) group, GR fluorescence intensity further decreased, GR nuclear translocation was markedly inhibited, and cytoplasmic GR distribution increased. ECGH nearly abolished Cort-induced GR nuclear translocation, similar to the effect of the GR antagonist RU486. These results indicate that ECG dose-dependently inhibits cort-induced GR upregulation and nuclear translocation in HSPCs (Figure 5B,C).

**Figure 6:**
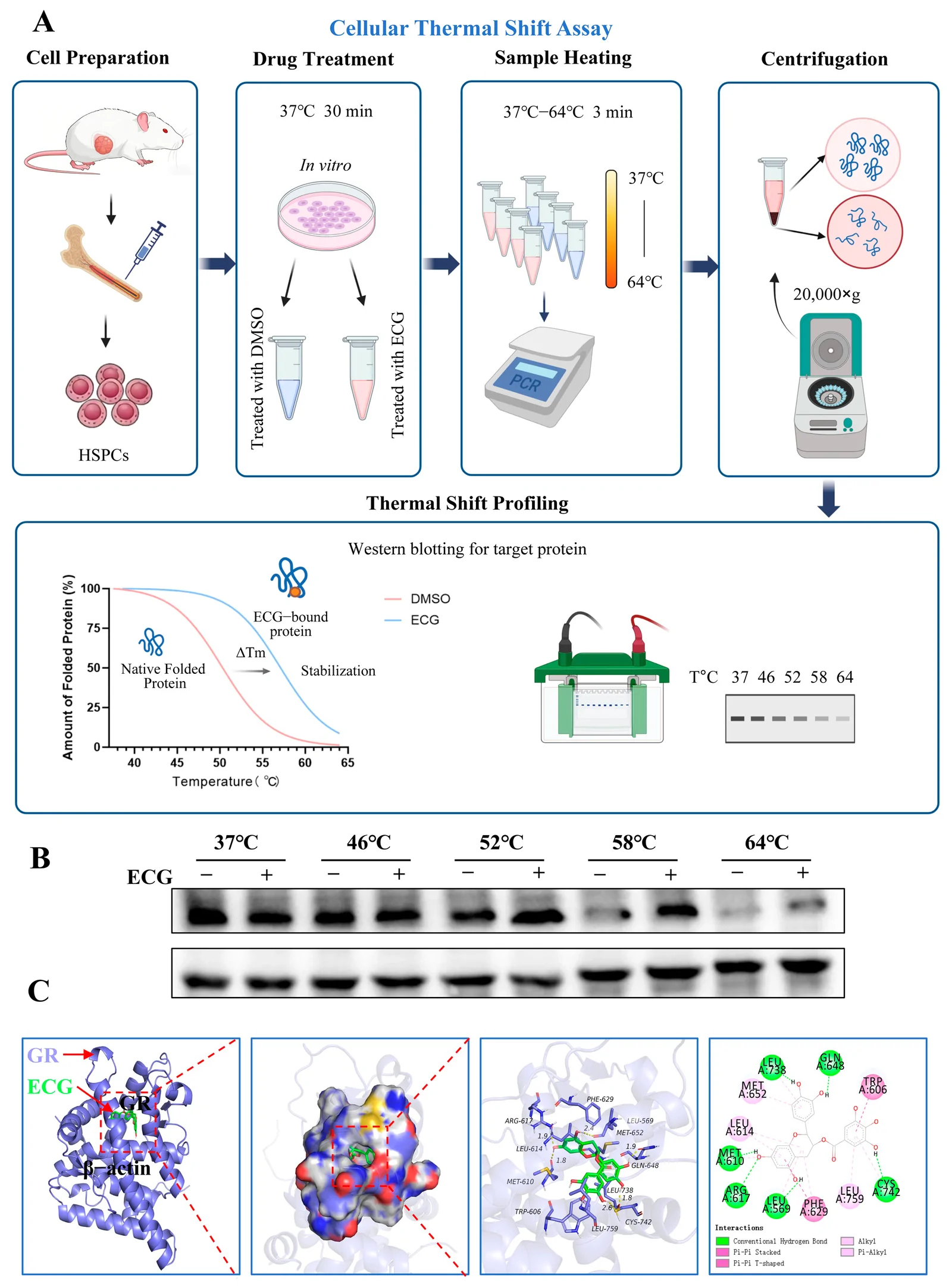
Validation of direct binding between epicatechin gallate (ECG) and glucocorticoid receptor (GR). HSPCs were treated with ECG (10 µM) for the cellular thermal shift assay coupled with Western blot (CETSA-WB). (**A**) CETSA-WB workflow. (**B**) CETSA-WB analysis of GR thermal stability. (**C**) Molecular docking of ECG into the GR binding pocket.

The direct interaction between ECG and GR was further validated. A CETSA-WB was performed on HSPC lysates to assess GR thermal stability upon ligand binding. Protein extracts were treated with 10 μM ECG or DMSO, followed by a CETSA heat pulse, after which soluble proteins were extracted and compared by Western blotting. The results showed that GR protein was significantly thermally stabilized in the EC-treated group, indicating that ECG may directly bind to GR (Figure 6A,B). Molecular docking results further predicted the binding sites between ECG and GR. The results showed that ECG bound to GR with a binding energy of –13.589 kcal/mol, and the interaction was primarily mediated by hydrogen bond networks and hydrophobic interactions, contributing to high-affinity binding. ECG formed eight hydrogen bonds with key residues within the active pocket of GR, with donor–acceptor distances ranging from 2.73 to 3.96 Å. In particular, the oxygen atoms of ECG served as acceptors forming hydrogen bonds with Leu614, Arg617, and Cys742, while acting as hydrogen bond donors to establish polar contacts with Leu569, Met610, Gln648, and Leu738. In addition, ECG engaged in hydrophobic interactions with residues including Leu569, Trp606, Met610, Leu614, Phe629, and Leu738, with binding distances all less than 4.0 Å (Figure 6C). These findings provide preliminary evidence that ECG may exert its pharmacological effects through direct binding to GR. We therefore speculate and predict that ECG may competitively bind to GR, ultimately inhibiting the aberrant differentiation of HSPCs into MDSCs under chronic stress conditions.

### 2.5. ECG Suppresses TNBC Metastasis Through the GR-HSPCs Axis

**Figure 7:**
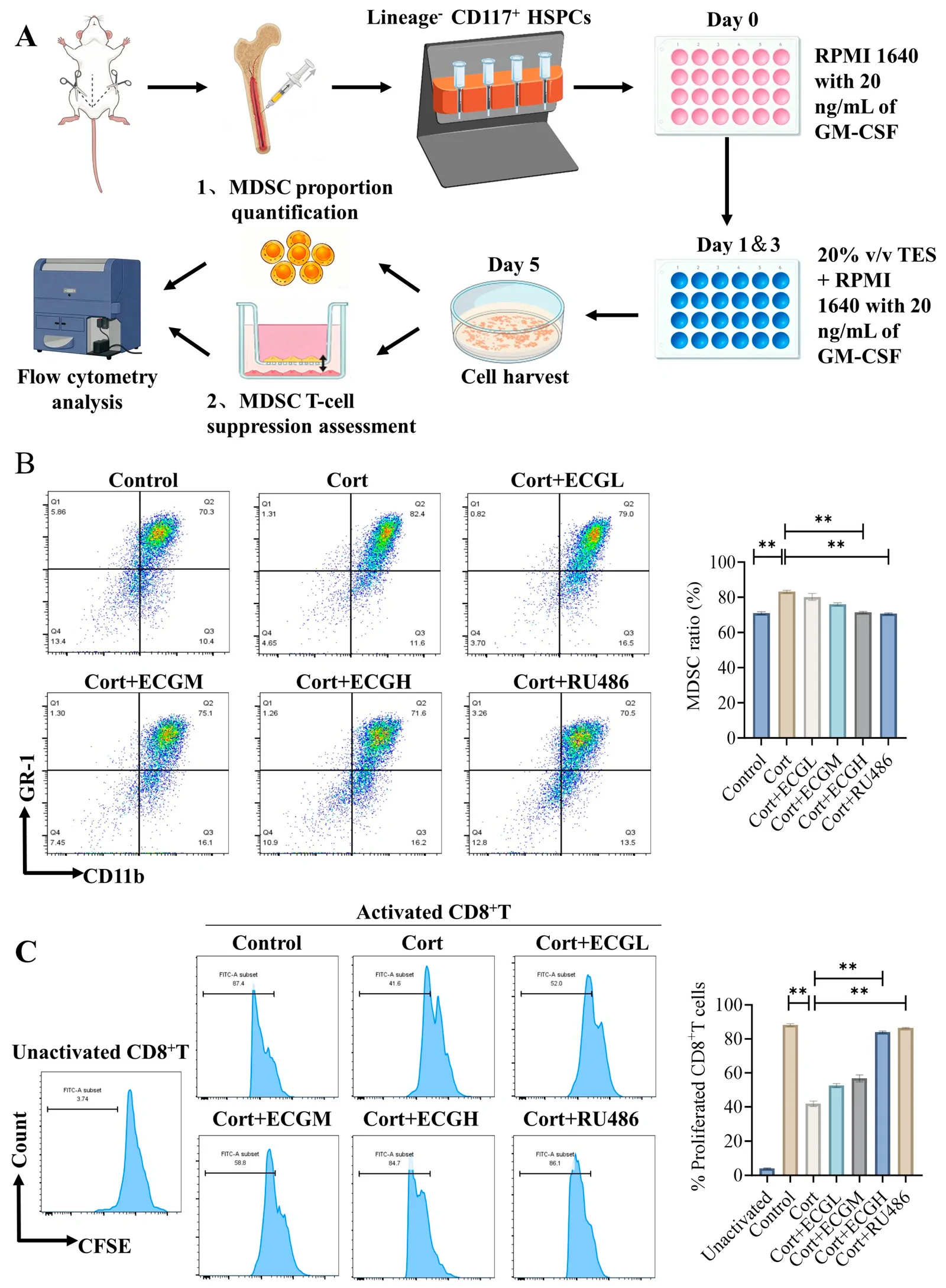
Epicatechin gallate (ECG) regulates hematopoietic stem and progenitor cell (HSPC) differentiation in a glucocorticoid receptor (GR)-dependent manner. HSPCs were treated with 200 nM corticosterone (Cort) alone or in combination with low-dose ECG (ECGL, 0.1 μM), medium-dose ECG (ECGM, 1 μM), high-dose ECG (ECGH, 10 μM), or RU486 (200 nM) for 24 h. (**A**) Schematic diagram of HSPC differentiation into myeloid-derived suppressor cells (MDSCs), followed by assessment of MDSC proliferation and immunosuppression. (**B**) Flow cytometric analysis of the effects of 200 nM Cort, various concentrations of ECG (0.1 μM, 1 μM, 10 μM), and RU486 (200 nM) treatment on the proportion of CD11b^+^Gr-1^+^ MDSCs derived from HSPCs. (**C**) CFSE assay detecting CD8^+^ T cell proliferation under the same treatment conditions. Data are presented as mean ± standard deviation (SD). ** *p* < 0.01.

GR is essential for MDSC differentiation, as genetic intervention experiments targeting GR in HSPCs showed that GR knockdown significantly reduced the MDSC proportion while GR overexpression increased it (Supplementary Figure S1B). We examined whether ECG inhibits breast cancer metastasis by regulating GR-dependent differentiation of HSPCs into MDSCs. HSPCs isolated from the bone marrow of tumor-bearing mice were first induced to undergo myeloid differentiation and then divided into the following groups: Control, Cort (200 nM), Cort + ECGL (0.1 μM), Cort + ECGM (1 μM), Cort + ECGH (10 μM), and Cort + RU486 (200 nM). Subsequently, the proportion of MDSCs (CD11b^+^Gr-1^+^) was assessed by flow cytometry to determine the effect of ECG on HSPCs differentiation (Figure 7A). The results showed that compared with the Control group, the proportion of MDSCs was significantly increased in the Cort treatment group, indicating that chronic stress markedly promoted the differentiation of HSPCs into MDSCs. ECG intervention effectively blocked this differentiation process. The proportion of MDSCs was significantly reduced in the Cort + ECGL group compared with the Cort group, and an even more pronounced reduction was observed in the Cort + ECGH group, indicating a dose-dependent effect. Meanwhile, the proportion of MDSCs was also significantly decreased in the Cort + RU486 group compared with the Cort group, with an effect comparable to that of the ECGH group (Figure 7B). Furthermore, CD8^+^ T cells were isolated from mouse spleens, CFSE-labeled, and co-cultured with supernatant from HSPC-derived MDSCs under anti-CD3/CD28 stimulation for 72 h. Consistent with the above results, Cort significantly inhibited T cell proliferation, while RU486 reversed this effect. ECG dose-dependently ameliorated the Cort-induced inhibition, with the high concentration mimicking RU486 (Figure 7C). These findings suggest that ECG suppresses the differentiation of HSPCs into MDSCs under chronic stress conditions in a GR-dependent manner, thereby reducing MDSC generation at the source and subsequently remodeling the breast cancer immune microenvironment.

**Figure 8:**
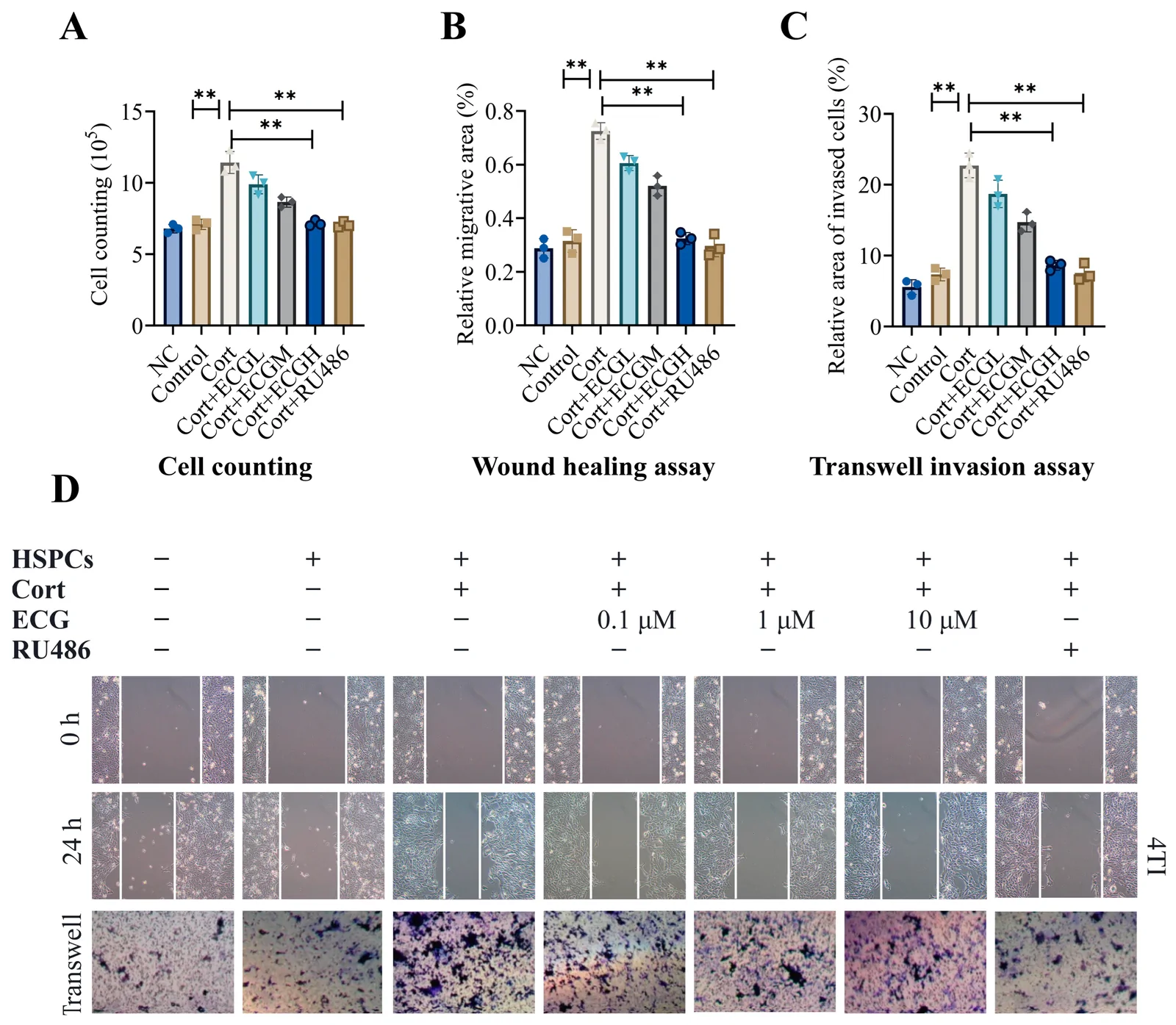
Effects of the indicated treatment on the proliferation, migration, and invasion of 4T1 mouse breast cancer cells in vitro. HSPCs were differentiated into MDSCs under different treatment conditions: vehicle, 200 nM corticosterone (Cort) alone, Cort plus low-dose ECG (ECGL, 0.1 μM), Cort plus medium-dose ECG (ECGM, 1 μM), Cort plus high-dose ECG (ECGH, 10 μM), or Cort plus RU486 (200 nM) for 24 h. Conditioned media from these MDSC cultures were then collected and used to treat 4T1 cells. (**A**) Cell counting assay for 4T1 cell proliferation (*n* = 3). (**B**) Quantification of the scratch wound healing assay (*n* = 3). (**C**) Quantification of the Transwell assay (*n* = 3). (**D**) Representative images of the scratch wound healing and Transwell assays. Data are presented as mean ± standard deviation (SD). ** *p* < 0.01.

**Figure 9:**
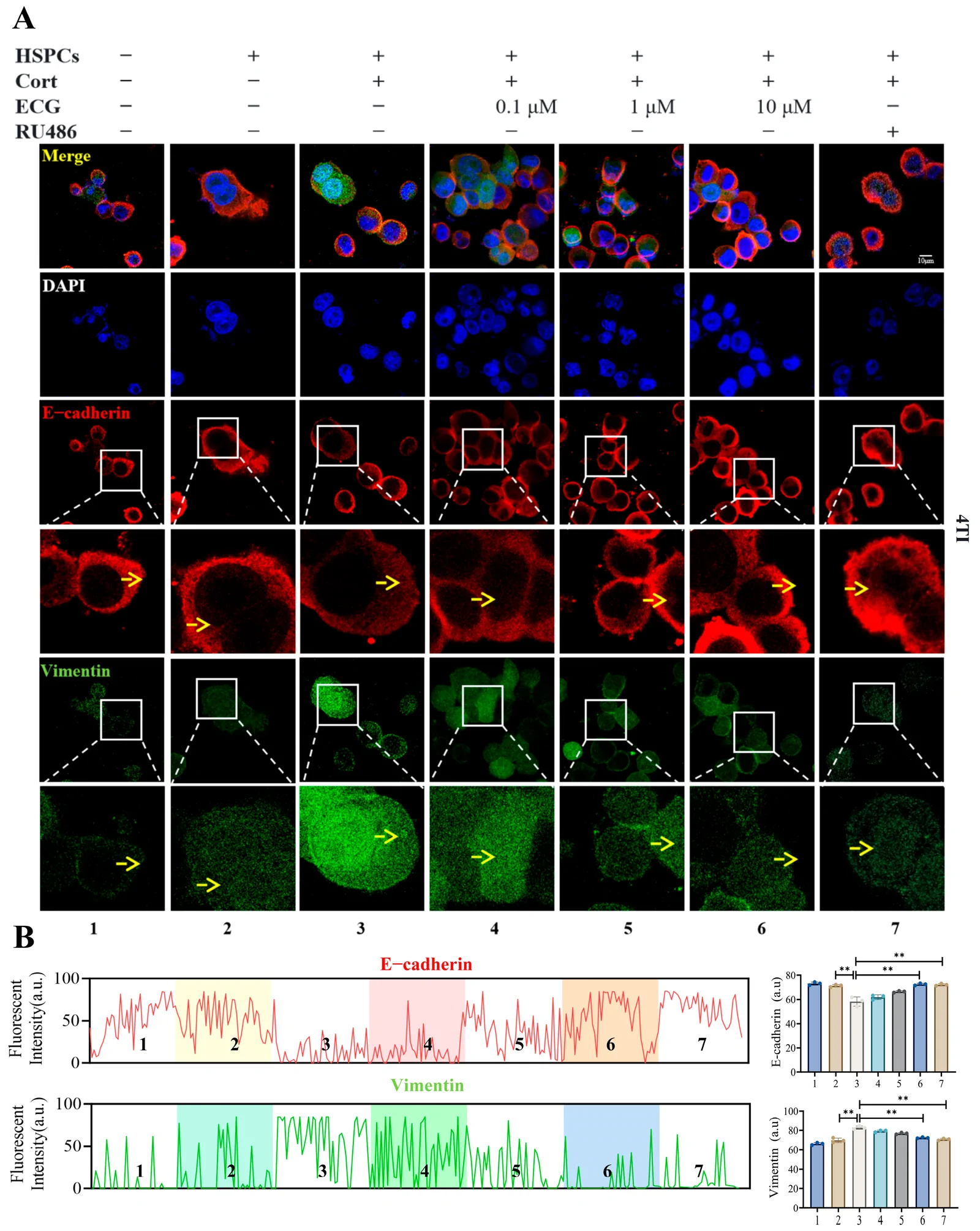
Effects of the indicated treatments on epithelial–mesenchymal transition (EMT) markers vimentin and E-cadherin in 4T1 mouse breast cancer cells. HSPCs were differentiated into MDSCs under different treatment conditions: vehicle, 200 nM corticosterone (Cort) alone, Cort plus low-dose ECG (ECGL, 0.1 μM), Cort plus medium-dose ECG (ECGM, 1 μM), Cort plus high-dose ECG (ECGH, 10 μM), or Cort plus RU486 (200 nM). Conditioned media were collected and applied to 4T1 cells. (**A**) Immunofluorescence detection of vimentin and E-cadherin expression in treated 4T1 cells. Blue: DAPI; Red: E-cadherin; Green: vimentin. (**B**) Left: fluorescence intensity line-scan analysis along the yellow dashed line. Scale bar: 10 μm. Right: Quantitative analysis of fluorescence intensity images (*n* = 3). Data are presented as the mean ± standard deviation (SD). ** *p* < 0.01.

To further determine whether ECG regulates HSPC-to-MDSC differentiation and thereby affects TNBC metastasis, HSPCs were induced to differentiate into MDSCs under various treatment conditions. The resulting MDSCs were then co-cultured with 4T1 breast cancer cells. Conditioned medium from MDSCs derived from Cort-treated HSPCs significantly promoted the proliferation, migration, and invasion of 4T1 cells compared with the control group. ECG treatment reversed this pro-tumor effect in a dose-dependent manner. Moreover, the GR antagonist RU486 exhibited an effect similar to that of ECGH (Figure 8A–D). The expression of EMT markers, including the epithelial marker E-cadherin and the mesenchymal marker vimentin, was also assessed by immunofluorescence. Conditioned medium from MDSCs derived from HSPCs (HSPC-induced MDSCs) led to decreased E-cadherin and increased vimentin expression in 4T1 cells compared with the control group, indicating enhanced metastatic potential. ECG treatment reversed these changes in a dose-dependent manner, with the ECGH group showing the most pronounced restoration of both markers. The GR antagonist RU486 produced effects similar to those of ECGH (Figure 9A). These findings indicate that ECG suppresses the myeloid differentiation of HSPCs into MDSCs under chronic stress conditions in a GR-dependent manner, thereby blocking the metastatic progression of breast cancer cells.

## 3. Discussion

HSPCs are emerging as both predictive biomarkers and therapeutic targets in breast cancer metastasis. Clinical studies have shown that elevated levels of HSPCs in the peripheral blood of breast cancer patients are associated with an increased risk of metastasis [23]. Under tumor-bearing conditions, tumor-derived cytokines such as granulocyte colony-stimulating factor (G-CSF) and granulocyte-macrophage colony-stimulating factor (GM-CSF) mobilize HSPCs from the bone marrow to metastatic organs (e.g., the lungs) [23], where they differentiate into immunosuppressive MDSCs, thereby supporting tumor cell colonization and outgrowth [24]. In the present study, we show that chronic psychological stress elevates circulating corticosterone levels, activates GR signaling in bone marrow-derived HSPCs, and biases their differentiation toward the MDSC lineage. Given the short lifespan of MDSCs, their continuous replenishment from HSPCs is critical for sustaining immunosuppression. These MDSCs extensively infiltrate lung tissue, upregulate hallmark molecules such as S100A8/A9, and suppress CD8^+^ T cell infiltration and activity, ultimately creating a pro-metastatic microenvironment. Thus, chronic stress promotes breast cancer lung metastasis at least in part through GR-dependent skewing of HSPC differentiation toward MDSCs.

Our research focuses on GC/GR-mediated HSPC activation by chronic psychological stress, but how signaling mediates HSPC-to-MDSC differentiation remains unclear. Retrospectively, potential interventional strategies to counteract this process can be broadly categorized into four aspects: firstly, by inhibiting GR nuclear translocation and thereby blocking the transcriptional activation of MDSC signature genes such as S100A8/A9, arginase-1 (Arg-1), and inducible nitric oxide synthase (iNOS) [25]; secondly, by interfering with the interaction between GR and myeloid transcription factors such as CCAAT/enhancer-binding protein beta (C/EBPβ) and SPI1 proto-oncogene transcription factor (PU.1), thereby restoring the differentiation balance of HSPCs [26,27]; thirdly, by blocking GR-mediated non-genomic signaling pathways, including PI3K/Akt and mitogen-activated protein kinase/extracellular signal-regulated kinase (MAPK/ERK), to suppress aberrant proliferation and myeloid differentiation of HSPCs; and fourthly, by reversing GR-driven epigenetic remodeling, thereby reducing the “pre-primed” state of HSPCs in response to differentiation signals within the tumor microenvironment [28]. The above potential mechanisms remain to be further validated in our subsequent experiments.

Targeting GR signaling to block tumor metastasis represents a potential strategy, yet conventional synthetic GR antagonists like RU486 are clinically limited by poor specificity and significant systemic side effects [29]. RU486 is a known dual GR/PR antagonist, with in vitro IC50 values of approximately 0.2 nM for PR and 2.6 nM for GR. Although its affinity for PR is higher, it still potently antagonizes GR in vivo, readily causing endocrine disorders, metabolic abnormalities, and other adverse reactions, rendering it unsuitable for long-term use in cancer patients [30]. In contrast, ECG, a natural GR signaling blocker, has been shown in this study to suppress chronic stress-induced MDSC generation at the source and inhibit breast cancer lung metastasis. Unlike conventional strategies that directly eliminate mature MDSCs, ECG acts upstream of HSPC differentiation, reducing MDSC production from the very beginning. If ECG exhibits high selectivity for GR without significant binding to PR, it may provide a safer GR-targeting strategy and avoid the adverse endocrine-disrupting effects of RU486. However, we currently lack direct evidence for the interaction between ECG and PR. Although molecular docking suggests that ECG forms a specific hydrogen bond network and hydrophobic stacking with key residues in the GR active pocket (e.g., Leu569, Arg617, Cys742, etc.), PR and GR share high homology in the ligand-binding domain. Therefore, ECG may theoretically bind to PR, and our preliminary data do not exclude this cross-reactivity. To distinguish ECG from RU486, our follow-up experiments will compare the binding characteristics of ECG to recombinant GR and PR, including competitive fluorescence polarization assays and SPR analysis for both receptors. Despite the unresolved issue of potential PR cross-reactivity—a key distinction from RU486 requiring further study—ECG offers notable translational advantages: it is biocompatible and low-toxic [31], and in our study it did not significantly affect body weight, supporting its safety for long-term adjuvant therapy without compromising quality of life. Its action is target-specific and dose-dependent, directly binding GR, blocking nuclear translocation, and inhibiting HSPC differentiation and metastasis, which informs clinical dosing. Furthermore, the target population is well defined: patients with chronic psychological stress (a condition common in lung and breast cancers) exhibit elevated glucocorticoids and aberrant GR signaling, and thus may benefit from ECG-based therapy.

Additionally, ECG inhibits epithelial–mesenchymal transition (EMT) and extracellular matrix remodeling, suggesting potential efficacy against other highly metastatic tumors, but this remains speculative, as our anti-metastatic findings were limited to a breast cancer lung metastasis model, and other tumor types or metastatic sites have not been tested. Mechanistically, the conformational changes induced by ECG binding and its interactions with coactivators/corepressors remain unclear. Moreover, the translational gap regarding ECG pharmacokinetics must be acknowledged: its oral bioavailability in humans is limited and not well established, and the doses used in our mouse study (25–50 mg/kg) would require allometric scaling and pharmacokinetic modeling (e.g., physiologically based pharmacokinetic (PBPK) before human dose estimation. Pharmacokinetic parameters, optimal administration route, and long-term safety all require systematic investigation. Despite these limitations, ECG’s unique upstream regulatory mechanism and favorable safety profile make it a promising adjuvant therapy option for breast cancer patients with stress-associated metastasis.

## 4. Materials and Methods

### 4.1. Experimental Reagent

Drugs: ECG (catalog No. 1257-08-5, HPLC ≥ 98%) was purchased from Vicchi (Chengdu, China). ECG was dissolved in Dimethyl sulfoxide (DMSO, catalog No. 67-68-5, purchased from Guanghua Technology (Guangzhou, China)) to prepare a 10 mM stock solution, which was aliquoted and stored at −20 °C protected from light. Hydrocortisone (a GR agonist, catalog No. HY-N0583/CS-2226, HPLC ≥ 99%) was purchased from MCE (Princeton, NJ, USA), and stock solutions were prepared in DMSO for in vitro experiments. RU486 (Mifepristone, a GR antagonist, catalog No. M830038-1g, HPLC ≥ 98%) was purchased from Macklin (Shanghai, China) and dissolved in DMSO for in vitro experiments.

Cell Culture: The mouse TNBC 4T1 cell line was purchased from Nanjing Kaiji Biotechnology Co., Ltd. (Nanjing, China). Fetal bovine serum (FBS, catalog No. 164210-50) was obtained from Procell (Wuhan, China). Penicillin-streptomycin (PS, catalog No. 15140-122), DMEM medium (catalog No. C11995500BT), RPMI-1640 medium (catalog No. C11875500BT), trypsin-EDTA (catalog No. 25200072), and phosphate-buffered saline (PBS, catalog No. C10010500BT) were purchased from Gibco (New York, NY, USA).

Establishment of the Tumor-Bearing Mouse Model: High-concentration Matrigel (catalog No. 082724) was purchased from Aibo Biotechnology (Shanghai, China).

*Histological Staining:* Paraformaldehyde/general tissue fixative (catalog No. BL539A) was purchased from NCM Biotech (Suzhou, China). The H&E staining kit (catalog No. DH0100) was obtained from Ligen (Beijing, China). Sodium citrate antigen retrieval solution (catalog No. C1031) and neutral balsam (catalog No. G8590) were purchased from Solarbio (Beijing, China). The two-step detection kit (catalog No. PV-9000) and DAB chromogen detection kit (catalog No. ZLI-9018) were obtained from ZSGB-BIO (Beijing, China).

Primary antibodies for IHC staining: The following antibodies were used: Ki67 antibody (catalog No. AF0198) was purchased from Affinity (Suzhou, China); GR antibody (catalog No. 24050-1-AP), fibronectin antibody (catalog No. 66042-1-Ig), MMP-2 antibody (catalog No. 66366-1-Ig), and CD8 antibody (catalog No. 85977-4-RR) were obtained from Proteintech (Wuhan, China); S100A8 antibody (catalog No. AF7929) and S100A9 antibody (catalog No. AF7932) were purchased from Beyotime Biotechnology (Shanghai, China).

ELISA: The cortisol ELISA kit (catalog No. MM-0565M1) was purchased from Meimian Company (Yancheng, China).

Immunofluorescence: Goat serum (catalog No. C0265) and DAPI staining solution (catalog No. C1006) were purchased from Beyotime Biotechnology (Shanghai, China). Triton X-100 (catalog No. 93443) was obtained from Sigma (Darmstadt, Germany). The primary antibodies used for detecting MDSC populations in lung tissues were as follows: Ly-6G/Ly-6C mouse antibody (catalog No. 14-5931-82) was purchased from Invitrogen (Shanghai, China), and CD11b rabbit antibody (catalog No. 31745-1-AP) was obtained from Proteintech (Wuhan, China). The primary antibody used for detecting GR expression and localization was as follows: GR rabbit antibody (catalog No. 24050-1-AP) was purchased from Proteintech (Wuhan, China). The primary antibodies used to assess the effect of HSPCs on the metastatic potential of 4T1 TNBC cells before and after treatment were as follows: Vimentin mouse antibody (catalog No. 60330-1-Ig) and E-cadherin rabbit antibody (catalog No. 20874-1-AP) were both purchased from Proteintech (Wuhan, China). ABflo^®^ 488-conjugated anti-mouse antibody (catalog No. AS037) and ABflo^®^ 555-conjugated anti-rabbit antibody (catalog No. AS058) were obtained from Abclonal (Wuhan, China); BF488-conjugated anti-rat antibody (catalog No. bs-0293G-BF488) was purchased from Bioss (Beijing, China). CoraLite 488-conjugated anti-rabbit antibody (catalog No. SA00013-2) was obtained from Proteintech (Wuhan, China).

CETSA-WB assay: The rabbit anti-GR antibody (catalog No. 24050-1-AP), mouse anti-β-actin antibody (catalog No. 66009-1-Ig), anti-mouse IgG secondary antibody (catalog No. SA00001-1), and anti-rabbit IgG secondary antibody (catalog No. SA00001-2) were purchased from Proteintech (Wuhan, China).

Plasmid DNA Transfection and siRNA Interference: The Nr3c1 plasmid and its vector plasmid were purchased from Guangzhou Dahong Biotechnology (Guangzhou, China), and the VGF transfection reagent was purchased from WZ Biosciences (Jinan, China). The siRNA targeting Nr3c1(m) and the siCtrl were purchased from Dahong Biosciences (Guangzhou, China), and the GP-transfect-Mate reagent was purchased from Genepharma (Shanghai, China).

Isolation and Identification of Mouse Bone Marrow HSPCs: The lineage cell depletion kit (catalog No. 130-090-858) was purchased from Miltenyi Biotec (Guangzhou, China). The CD117 microbeads (catalog No. 130-091-224) were purchased from Miltenyi Biotec (Guangzhou, China). FITC-conjugated mouse Sca-1 antibody and PE-conjugated mouse c-Kit antibody.

Flow Cytometric Analysis of MDSC differentiation: GM-CSF (TargetMol, TMPY-03662, Shanghai, China), FITC-conjugated anti-mouse CD11b antibody (BioLegend, 101205, San Diego, CA, USA) and APC-conjugated anti-mouse GR-1 antibody (BioLegend, 108411, San Diego, CA, USA).

CFSE detection assay: The mouse CD8^+^ T cell isolation kit (catalog No. B90010) was purchased from Selleck (Houston, TX, USA). The CFSE Cell Proliferation and Tracking Kit (catalog No. 423801) was purchased from Biolegend (San Diego, CA, USA). The anti-mouse CD3 antibody (catalog No. A2104) was purchased from Selleck (Houston, TX, USA). The anti-mouse CD28 antibody (catalog No. A2166) was purchased from Selleck (Houston, TX, USA). The IL-2 cytokine (catalog No. 212-12-20UG) was purchased from PeproTech (Cranbury, NJ, USA).

### 4.2. Cell Culture

Mouse TNBC 4T1 cells were cultured in DMEM medium supplemented with 10% FBS and 1% PS, and maintained in a constant temperature and humidity incubator at 37 °C with 5% CO_2_ for routine subculture. Cells in the logarithmic growth phase were used for subsequent experiments.

### 4.3. Animal Experimentation

Five-week-old specific pathogen-free female BALB/c mice were purchased from the Guangdong Provincial Animal Center. All animal experimental protocols were reviewed and approved by the Experimental Animal Ethics Committee of Guangzhou University of Chinese Medicine. After one week of acclimatization, the mice were randomly divided into four groups: Control group, CUMS group, CUMS + ECGL group, and CUMS + ECGH group. The modified CUMS protocol was used to induce depressive-like behaviors in mice [32]. All behavioral tests and metastatic nodule counting were conducted by investigators blinded to the group allocation. After 7 days of stress pretreatment, 4T1 cells were collected, mixed with Matrigel at a 1:1 volume ratio, and orthotopically inoculated into the mammary fat pad of mice at a density of 2 × 10^6^ cells per mouse. Stress treatment was continued after inoculation until the experimental endpoint. ECG was administered by gavage daily before stress exposure, with doses of 25 mg/kg and 50 mg/kg for the low-dose and ECGH groups, respectively [33]. The control and CUMS groups received an equal volume of normal saline by gavage.

### 4.4. H&E Staining

Lung tissues from each group of mice were collected and fixed in 4% paraformaldehyde for 24 h. After dehydration through a graded ethanol series, clearing in xylene, and embedding in paraffin, tissue sections were cut at a thickness of 4 μm. The sections were deparaffinized, rehydrated, and stained with hematoxylin, followed by rinsing and counterstaining with eosin. After subsequent dehydration, clearing, and mounting, the sections were observed and images were captured using an optical microscope.

### 4.5. IHC Staining

Tissue sections were immersed in citrate buffer and boiled for 2 min for antigen retrieval, then allowed to cool naturally to room temperature and washed with PBS. Endogenous peroxidase activity was blocked by incubating the sections in hydrogen peroxide solution at room temperature for 10 min, followed by three washes with PBS. Then the sections were incubated with primary antibodies at 4 °C overnight. The primary antibodies used were as follows: Ki67 antibody (1:200), GR antibody (1:200), S100A8 antibody (1:100), S100A9 antibody (1:100), fibronectin antibody (1:300), MMP-2 antibody (1:200), and CD8 antibody (1:500). After three washes with PBS, the sections were incubated with the corresponding secondary antibodies at room temperature for 1 h. Following three additional washes with PBS, color development was performed using DAB for 5 min. The sections were then counterstained with hematoxylin, dehydrated through a graded ethanol series, cleared in xylene, and mounted with neutral balsam. Images were captured using an optical microscope.

### 4.6. ELISA

Serum cortisol levels were detected by ELISA, which was carried out strictly in accordance with the manufacturer’s instructions. Specifically, mouse serum samples were mixed with the antibody working solution and incubated at 37 °C for 30 min. After washing the microplate, the enzyme conjugate working solution was added, and the plate was incubated at 37 °C for 30 min. The substrate solution was then added for color development. Following a 10 min incubation at 37 °C, the stop solution was added to terminate the reaction. The absorbance (optical density, OD) was measured at 450 nm using a microplate reader, and the cortisol concentration was calculated based on the standard curve.

### 4.7. Immunofluorescence

Detection of MDSCs in lung metastatic tissue: The tissue sections were blocked with 5% goat serum at room temperature for 1 h, after which the blocking solution was discarded. The sections were incubated overnight at 4 °C with a mixture of primary antibodies containing Ly-6G/Ly-6C mouse antibody (1:200) and CD11b rabbit antibody (1:200). The following day, the sections were incubated for 2 h at room temperature with a mixture of secondary antibodies consisting of BF488-conjugated anti-rat antibody and Alexa Fluor^®^ 555-conjugated anti-rabbit antibody. Nuclei were counterstained with DAPI, and after mounting with an anti-fade mounting medium, images were captured using a laser scanning confocal microscope.

Detection of GR expression and localization: Treated HSPCs were incubated overnight at 4 °C with GR rabbit antibody (1:200), followed by incubation for 2 h with CoraLite 488-conjugated anti-rabbit secondary antibody. Nuclei were counterstained with DAPI, and after mounting, images were captured using a laser scanning confocal microscope.

Effect of drug-containing supernatant from differentiated HSPCs on the metastatic potential of 4T1 cells: Treated 4T1 cells were seeded at a density of 1 × 10^6^ cells/well into 24-well plates containing glass coverslips. After cell adhesion, cells were fixed with 4% paraformaldehyde at room temperature for 10 min, permeabilized with 0.5% Triton X-100 at room temperature for 15 min, and blocked with 5% goat serum at room temperature for 1 h. Cells were incubated overnight at 4 °C with vimentin mouse antibody (1:500) and E-cadherin rabbit antibody (1:200) primary antibodies. The following day, cells were incubated for 2 h at room temperature with a mixture of secondary antibodies consisting of Alexa Fluor^®^ 488-conjugated anti-mouse antibody and Alexa Fluor^®^ 555-conjugated anti-rabbit antibody. Nuclei were counterstained with DAPI, and after mounting, images were captured using a laser scanning confocal microscope.

ImageJ (v1.54q) was used to analyze the resulting images. For qualitative analysis, a line was drawn along the direction of analysis. The fluorescence intensity profile was obtained using Plot Profile. The data were exported to Excel. Graphs could then be created in GraphPad Prism (v10.1.2). To keep multiple graphs consistent, the line was saved in the ROI Manager and reused. Dashed line markers could be added optionally. Quantitative analysis included measurements of nuclear fluorescence intensity and colocalization area. To measure nuclear fluorescence intensity, split the channels or convert the image to 8-bit. Adjust the threshold on the nuclear channel to create a selection. Add the selection to the ROI Manager. Then measure the integrated density of the target channel. To measure the colocalization area, split the channels and delete the blue DAPI channel. Keep the red and green channels. Use the Image Calculator. Apply a uniform threshold. Choose “Area fraction” and measure.

### 4.8. Molecular Docking

The molecular structure of the compound (ECG) was obtained from the PubChem database (https://pubchem.ncbi.nlm.nih.gov/ (accessed on 7 December 2025)). Format conversion and energy minimization were performed using Chem3D, and the compound was imported into Schrödinger 2019.01 software to establish a database. Following hydrogen addition, structural optimization, and energy minimization, the compound was saved as the ligand for molecular docking. The protein structure of GR (PDB ID: 8W03) was obtained from the RCSB Protein Data Bank (https://www.rcsb.org/ (accessed on 7 December 2025)). The protein structure was processed on the Maestro 11.9 platform using the Protein Preparation Wizard module in Schrödinger 2019.01. Crystallographic water molecules were removed, missing hydrogen atoms were added, bond order information was corrected, and missing peptide segments were repaired. The protein was then subjected to energy minimization and geometric structure optimization [34]. Molecular docking was performed using the Glide module in Schrödinger 2019.01. Protein processing was carried out using the Protein Preparation Wizard module, which involved preprocessing, optimization, and minimization of the receptor (constrained minimization using the OPLS3e force field). All molecules were prepared following the default settings of the LigPrep module. During screening in the Glide module, the prepared receptor was imported, and the appropriate binding site was specified in receptor grid generation. The native ligand of the protein was selected and used as the centroid of a 15 Å × 15 Å × 15 Å grid box (coordinates: x = −33.62, y = 14.36, z = 29.53). Molecular docking and screening were performed using the induced fit docking method. The interaction mode between the compound and the target protein was analyzed, including interactions between the compound and protein residues, such as hydrogen bonding, π–π stacking, covalent interactions, and hydrophobic interactions. Finally, based on the docking scores of the compounds, the potential activity of the candidate compound was evaluated.

### 4.9. CETSA-WB

The CETSA-WB was conducted as previously described [35]. Briefly, HSPCs were collected and lysed to obtain soluble protein lysate. The lysate was aliquoted into PCR tubes and treated with 10 μM ECG or DMSO at 37 °C for 30 min prior to the CETSA heat pulse. The samples were heated at the indicated temperatures (37 °C, 46 °C, 52 °C, 58 °C, and 64 °C) for 3 min, followed by cooling at 4 °C for 3 min in a thermocycler (Applied Biosystems, Waltham, MA, USA). After centrifugation at 20,000× *g* for 20 min at 4 °C, the soluble supernatants were subjected to Western blotting. Primary antibodies were rabbit anti-GR and mouse ant-β-actin, while secondary antibodies were anti-mouse IgG and anti-rabbit IgG.

### 4.10. Plasmid DNA Transfection and siRNA Interference

For plasmid DNA transfection, either the Nr3c1 plasmid or its vector plasmid was transfected at 1 μg per well into cells in 12-well plates (1 × 10^5^ cells per well) using VGF transfection reagent according to the manufacturer’s instructions. After 48 h, cells were collected for subsequent molecular analyses.

For siRNA interference, siRNA targeting either Nr3c1(m) or siCtrl was transfected using GP-transfect-Mate followed by incubation at 37 °C for 48 h. The siRNA sequence for Nr3c1(m) was 5′-GGUGUUAUAUGCAGGAUAUTT-3′, and the siCtrl sequence was 5′-UUCUCCGAACGUGUCACGUTT-3′.

### 4.11. Isolation and Identification of Mouse Bone Marrow HSPCs

HSPC isolation was performed according to the manufacturer’s instructions using the lineage cell depletion kit and CD117 microbeads. Under sterile conditions, the femurs and tibias of mice were collected. Both ends of the bones were cut off, and the bone marrow cavity was repeatedly flushed with pre-chilled sterile PBS using a syringe to prepare a single-cell suspension of bone marrow cells. The suspension was filtered through a 70 μm cell strainer to remove tissue debris. Red blood cell lysis buffer was added for 5 min at room temperature to lyse erythrocytes, followed by centrifugation at 300× *g* for 5 min. The supernatant was discarded, and the cell pellet was collected. Lineage-negative (Lin^−^) cells were isolated by MACS according to the manufacturer’s instructions for the mouse lineage cell depletion kit. Lin^−^ cells were then incubated with CD117 (c-Kit) microbeads at 4 °C for 15 min, followed by a second MACS separation to obtain c-Kit^+^ cells. After staining, cell purity was assessed using a BD Biosciences flow cytometer to validate the efficiency of HSPC isolation. The Lin^−^c-Kit^+^ cells were immunolabeled with FITC-conjugated mouse Sca-1 antibody and PE-conjugated mouse c-Kit antibody.

### 4.12. Flow Cytometric Analysis of MDSC Differentiation

The process was carried out following the manufacturer’s instructions and previously published studies [36]. HSPCs isolated by magnetic bead sorting were resuspended in complete RPMI 1640 medium and seeded into 24-well plates at a density of 25,000 cells/mL. On Day 0, GM-CSF was added to the medium at a final concentration of 20 ng/mL, followed by drug treatment for 24 h. The Control group received no drug treatment. The CUMS simulation group was treated with 200 nM Cort. ECG intervention groups were treated with 200 nM Cort combined with 0.1 μM, 1 μM, and 10 μM ECG, respectively. The positive Control group was treated with 200 nM Cort combined with 200 nM RU486. On Day 1 and Day 3, 20% volume of tumor tissue supernatant (TES) prepared from tumor-bearing mice was supplemented into each well, together with complete RPMI 1640 medium containing 20 ng/mL GM-CSF. On Day 5, cells from each group were collected and resuspended in 100 μL of PBS at a density of 1 × 10^6^ cells. Immunostaining was performed using FITC-conjugated anti-mouse CD11b antibody and APC-conjugated anti-mouse Gr-1 antibody. Following staining, the cells were resuspended in PBS containing 2% FBS, and data acquisition and analysis were carried out using a BD Biosciences flow cytometer.

### 4.13. CFSE Detection

The CFSE detection was carried out following the manufacturer’s instructions and previously published studies [37]. In particular, mouse spleens were aseptically isolated and single-cell suspensions of splenocytes were prepared. CD8^+^ T cells were purified from the splenocyte suspensions using a mouse CD8^+^ T cell isolation kit. The purity of the isolated CD8^+^ T cells was assessed by flow cytometry, and cells with a purity ≥90% were used in subsequent experiments. The proliferation capacity of CD8^+^ T cells was evaluated using a CFSE Cell Proliferation and Tracking Kit. Purified CD8^+^ T cells were stained with CFSE at a final concentration of 2.5 μM for 20 min at 37 °C in the dark. Staining was terminated by adding RPMI 1640 medium supplemented with 10% FBS, followed by extensive washing to remove residual dye. The labeled CD8^+^ T cells were then activated in complete medium containing anti-mouse CD3 and CD28 antibodies and IL-2 cytokine, and simultaneously co-cultured with the supernatant of MDSCs differentiated from treated HSPCs for 72 h. Subsequently, the proliferation level of CD8^+^ T cells was assessed by flow cytometry based on CFSE fluorescence dilution.

### 4.14. Cell Proliferation, Migration, and Invasion Assays

For proliferation assessment, 4T1 cells were seeded into 6-well plates (3 × 10^5^ cells/well). HSPCs were pre-treated with Cort alone or with ECG or RU486, differentiated into MDSCs, and the resulting supernatants were collected. These supernatants were then added to 4T1 cells. After 24 h, 4T1 cells were counted. For the migration assay, confluent 4T1 monolayers were wounded with a pipette tip and cultured with the supernatants; images were taken after 24 h. For invasion detection, Transwell inserts (8 μm pores) pre-coated with Matrigel were used. 4T1 cells (1 × 10^5^/insert) in serum-free medium were seeded into the upper chamber, and the supernatants were placed in the lower chamber. After 24 h, cells were fixed with 4% paraformaldehyde and stained with crystal violet.

### 4.15. Statistical Analysis

All experiments in this study were independently repeated at least three times, and the experimental data are presented as mean ± standard deviation (SD). Statistical analysis was performed using SPSS 20.0 software. Comparisons among multiple groups were conducted using one-way analysis of variance. For post hoc multiple comparisons, the Bonferroni method was applied when variances were homogeneous, whereas Dunnett’s T3 test was used when data met the assumption of normal distribution but variances were unequal. A value of (*p* < 0.05) was considered statistically significant.

## 5. Conclusions

In conclusion, we demonstrate that ECG directly targets GR and competitively blocks its nuclear translocation. By inhibiting chronic stress-induced aberrant differentiation of HSPCs into MDSCs at the source, this intervention remodels the pulmonary immune microenvironment and effectively suppresses stress-promoted breast cancer lung metastasis. Our study reveals ECG as a natural GR signaling blocker that intervenes in stress-associated tumor metastasis, providing key experimental evidence and a candidate lead molecule for targeting the GR-HSPCs axis.

## Data Availability

The original contributions presented in this study are included in the article/Supplementary Material. Further inquiries can be directed to the corresponding authors.

## Supplementary Materials

The following supporting information can be downloaded at https://www.mdpi.com/article/10.3390/ph19081211/s1: Figure S1: GR regulates the differentiation of HSPCs into MDSCs. (A) Representative immunofluorescence images of GR in HSPCs under different treatments. Blue: DAPI (nuclei); Green: GR. Scale bar: 10 μm. No obvious differences in GR nuclear translocation were observed among groups under basal conditions without cortisol stimulation. (B) Flow cytometry contour plots and quantitative statistics showing the proportion of CD11b^+^GR-1^+^ MDSCs upon GR knockdown or overexpression in HSPCs. ** *p* < 0.01.

## Abbreviations

The following abbreviations are used in this manuscript:

TNBC	Triple-negative breast cancer
MDSCs	Myeloid-derived suppressor cells
HSPCs	Hematopoietic stem and progenitor cells
ECG	Epicatechin gallate
CUMS	Chronic unpredictable mild stress
GC	Glucocorticoid
GR	Glucocorticoid receptor
Cort	Corticosterone
FAK	Focal Adhesion Kinase
PD-L1	Programmed death-ligand 1
S100A8	S100 calcium-binding protein A8
S100A9	S100 calcium-binding protein A9
MMP-2	Matrix metalloproteinase-2
RAGE	Receptor for advanced glycation end-products
TLR4	Toll-like receptor 4
Arg-1	Arginase-1
iNOS	Inducible nitric oxide synthase
ACC	Acetyl-CoA carboxylase
ACLY	ATP-citrate lyase
FASN	Fatty acid synthase
PI3K	Phosphatidylinositol 3-kinase
AKT	Protein kinase B
mTOR	Mammalian target of rapamycin
TGF-β1	Transforming growth factor-β1
EMT	Epithelial–mesenchymal transition
uPA	Urokinase plasminogen activator
ER	Estrogen receptor
PR	Progesterone receptor
HER2	Human epidermal growth factor receptor 2
mOS	Median overall survival
HPA	Hypothalamic–pituitary–adrenal
SNS	Sympathetic nervous system
TME	Tumor microenvironment
HIF-1α	Hypoxia-inducible factor 1α
TCM	Traditional chinese medicine
MACS	Magnetic-activated cell sorting
TAMs	Tumor-associated macrophages
G-CSF	Granulocyte colony-stimulating factor
GM-CSF	Granulocyte-macrophage colony-stimulating factor
IL-6	Interleukin-6
STAT3	Signal transducer and activator of transcription 3
NF-κB	Nuclear factor-kappa B
CCR1	C-C chemokine receptor type 1
CCR5	C-C chemokine receptor type 5
CXCL1	C-X-C motif chemokine ligand 1
CXCR2	C-X-C chemokine receptor type 2
EZH2	Enhancer of zeste homolog 2
SMARCE1	SWI/SNF-related matrix-associated actin-dependent regulator of chromatin subfamily E member 1
C/EBPβ	CCAAT/enhancer-binding protein beta
PU.1	SPI1 proto-oncogene transcription factor
MAPK	Mitogen-activated protein kinase
ERK	Extracellular signal-regulated kinase
PBPK	Physiologically based pharmacokinetic
